# Dietary interventions in the management of atrial fibrillation

**DOI:** 10.3389/fcvm.2024.1418059

**Published:** 2024-08-01

**Authors:** Muhammad Ahad Nabil, Leanne Rychlik, Audrey Nicholson, Peter Cheung, Gregory D. Olsovsky, Jaime Molden, Ajay Tripuraneni, Shayan-Salehi Hajivandi, Javier E. Banchs

**Affiliations:** ^1^ Department of Medicine, Division of Cardiology, Baylor Scott & White Health, Round Rock, TX, United States; ^2^ Department of Medicine, Division of Cardiology, Baylor Scott & White Health, Temple, TX, United States

**Keywords:** atrial fibrillation, diet, nutrients, alcohol, caffeine, prevention

## Abstract

Atrial fibrillation (AF) represents the most common cardiac arrhythmia with significant morbidity and mortality implications. It is a common cause of hospital admissions, significantly impacts quality of life, increases morbidity and decreases life expectancy. Despite advancements in treatment options, prevalence of AF remains exceptionally high. AF is a challenging disease to manage, not just clinically but also financially. Evidence suggests lifestyle modification, including dietary changes, plays a significant role in the treatment of AF. This review aims to analyze the existing literature on the effects of dietary modifications on the incidence, progression, and outcomes of atrial fibrillation. It examines various dietary components, including alcohol, caffeine, omega-3 polyunsaturated fatty acids and minerals, and their impact on AF incidence, progression, and outcomes. The evidence surrounding the effects of dietary patterns, such as the Mediterranean and low carbohydrate diets, on AF is also evaluated. Overall, this review underscores the importance of dietary interventions as part of a comprehensive approach to AF management and highlights the need for further research in this emerging field.

## Introduction

Atrial fibrillation is the most common cardiac arrhythmia characterized by irregular and often rapid heartbeat (1). It is a common cause of hospital admissions with high readmission rates and significantly impacts quality of life (2). Patients with atrial fibrillation have an average reduction in their life expectancy and significant morbidity (3). Despite advancements in pharmacological therapy and catheter-based procedures, the prevalence of AF remains exceptionally high. There are many factors associated with AF, and the chance of spontaneous conversion back to sinus rhythm is lower with the existence of one or more of these risk factors, which include, but are not limited to, heart failure, left atrial size, and duration of the patient in AF (4, 5) As these risk factors are commonly present in the patients of modern times, the prevalence of AF remains high. It is a challenging disease to manage, not just clinically but also financially, as it places a tremendous burden on healthcare globally (2). Previous studies and emerging evidence suggest lifestyle changes, including dietary changes, play a significant role in preventing and managing AF (6) Its recurrent nature, associated symptoms, increased risk of stroke, heart failure, and overall cardiovascular morbidity and mortality demands significant attention and development of more effective management strategies. The current therapeutic approach primarily focuses on symptom and heart rate management via rate and/or rhythm control, in addition to stroke prevention with the implementation of anticoagulation or mechanical exclusion of the left atrial appendage. A growing body of evidence supports restoration and maintenance of sinus rhythm as the preferred management strategy (7). Limited success maintaining sinus rhythm long term, toxicity of the antiarrhythmic drugs, progressive nature of associated co-morbidities and limited understanding of the atrial pathophysiology involved in the natural history of AF, has made it difficult to halt or slow its progression. However, the association of alcohol intake, obesity, diabetes, and autonomic imbalance with the incidence, recurrence and progression of AF has been well-recognized for quite some time (6). More recently, there has been a growing body of research supporting the adoption of risk factor modification as the fourth pillar in the management of AF, as proposed in the latest revision of the clinical practice guidelines published by the American College of Cardiology and American Heart Association Joint Committee (8). Lifestyle modifications have demonstrated a significant impact on the prevention and recurrence of AF. Dietary changes are a significant part of these lifestyle modifications. This review aims to provide a thorough overview of the evidence supporting specific dietary modifications in managing AF.

## Methods

### Databases and search strategy

A comprehensive and systematic literature search was conducted in two major electronic databases: PubMed and the Cochrane Library. These databases were selected due to their extensive collections of medical and clinical research articles. The aim was to identify studies published up to 2024 that examined the relationship between dietary factors and atrial fibrillation.

### Keywords and search terms

The search strategy utilized a combination of keywords to ensure a broad and thorough search. Key terms included “Atrial fibrillation”, “Diet”, “Nutrition”, “Dietary interventions”. These terms were chosen to capture a wide range of studies that could provide insights into how diet and nutrition impact AF.

### Inclusion criteria

To ensure relevance and quality, studies had to meet the following inclusion criteria:

Population: Studies involved human participants diagnosed with AF or those at risk of developing the condition.
Publication: Only studies published in peer-reviewed journals were considered.

### Screening and selection process

The selection process involved several steps to ensure rigorous filtering and inclusion of relevant studies:

Initial Screening: Three independent reviewers screened the titles and abstracts of all identified articles. This initial step was crucial for excluding studies that were clearly irrelevant based on the title and abstract alone.
Full-Text Assessment: Articles that passed the initial screening were then subjected to a full-text assessment and the content of each of these studies was further investigated.
Discrepancy Resolution: Any discrepancies or disagreements among the reviewers were resolved through discussion and consensus, ensuring that the selection process was both thorough and unbiased.

### Data extraction and synthesis

Given the anticipated diversity in study designs, populations, dietary interventions, and outcomes, a narrative synthesis approach was adopted. This method involves summarizing and interpreting the findings of the included studies in a descriptive manner rather than relying solely on statistical analysis. The narrative synthesis allowed for identification of common themes and highlighting consistent findings across different studies, contextual analysis which helped in understanding how different dietary factors may influence AF in various contexts and integration of diverse evidence by combining results from studies with varying methodologies to provide a comprehensive overview.

### Summary of findings

The narrative synthesis aimed to collate and summarize the evidence regarding the impact of dietary interventions on atrial fibrillation. Key aspects included:

Types of dietary interventions: Examination of specific diets (e.g., Mediterranean diet), individual nutrients (e.g., omega-3 fatty acids), and other dietary components.
Outcomes: Analysis of outcomes such as AF incidence, symptom severity, and recurrence rates.
Population characteristics: Consideration of how different populations (e.g., age groups, comorbid conditions) respond to dietary changes.

By employing this systematic and structured approach, the review aimed to provide a detailed and comprehensive understanding of the current evidence on dietary interventions and their effects on atrial fibrillation.

## Diets, nutrients and atrial fibrillation

### Omega-3 fatty acids

The potential role of omega-3 fatty acids, primarily derived from fish consumption or supplementation, in influencing AF incidence has been extensively studied. Several lines of evidence from observational studies and randomized trials shed light on the complex relationship between omega-3 fatty acids and AF risk. Observational studies initially suggested a preventive effect of fish consumption against new-onset AF, particularly among elderly adults (9). Specifically, greater intake of broiled/baked fish, notably tuna, has been associated with a lower risk of AF. The protective effect is attributed to the beneficial impact of long-chain omega-3 fatty acids in fatty fish. However, fried fish or fish sandwiches did not confer the same protective effect, underscoring the importance of preparation methods (9). In contrast, randomized trials exploring marine omega-3 fatty acid supplementation for primary AF prevention have yielded discouraging results (10). A trial over 5.3 years found no significant effect in AF incidence compared to placebo, challenging the efficacy of omega-3 fatty acid supplements (10). In the other hand, a large cohort study involving over 54,000 participants over 13 years found that *in vivo* levels of omega-3 fatty acids had no association to the risk of incident AF, supporting their safety to regards to AF risk (11). A recent meta-analysis of prospective studies involving more than 200,000 participants and up to 12,000 cases of AF found no significant association between higher fish consumption or intake of omega-3 polyunsaturated fatty acids (PUFAs) and the development of AF (12). While fish and omega-3 PUFAs have been inversely associated with various cardiovascular diseases, including stroke and coronary heart disease, no significant inverse association was found with AF in this meta-analysis. The conflicting outcomes of clinical trials and observational studies may be attributed to various factors, including the heterogeneity of patient populations, differences in fish preparation methods, and the complex interplay between dietary components and cardiovascular health. Furthermore, the electrophysiological effects of omega-3 PUFAs, particularly EPA and DHA found in fish oil, on cardiac ion channels and membrane properties add another layer of complexity to the understanding of their potential role in arrhythmia prevention (13, 14). It is hypothesized that fatty acids may influence parameters crucial for generating and maintaining arrhythmias, but their impact on AF recurrence or postoperative AF, remains unclear. While omega-3 fatty acids derived from fish consumption or supplementation have been associated with various cardiovascular benefits, including potential anti-arrhythmic effects, currently they have no role in AF prevention or management. Further research and a higher level of evidence is necessary to better understand the association between omega-3 fatty acids, their modality of consumption, and cardiovascular health before their inclusion in strategies to treat or prevent AF.

### Mediterranean diet

The Mediterranean Diet (Med-Diet) characterized by a high consumption of fruits, vegetables, whole grains, legumes, nuts, seeds, olive oil, a modest intake of fish and poultry, and a low consumption of red meat and sweets (15) has emerged as a robust preventative strategy against the development of AF. Numerous studies have demonstrated that adherence to the Mediterranean diet reduces the risk of AF and other manifestations of cardiovascular disease (16). Paradoxically and despite the lack of definitive evidence in support of omega 3 fatty acids supplements, it has been postulated that the Mediterranean diet's emphasis on foods rich in omega-3 fatty acids may contribute to decrease inflammation and improve cardiac function, lowering the risk of AF. Another proposed mechanism is the Mediterranean diet's high intake of fruits, vegetables, and olive oil which provide ample antioxidants and polyphenols, which have anti-inflammatory and cardioprotective effects (16). Hence the benefit could be attributable to the latter or their combination. The PREDIMAR study investigated the efficacy of a remotely delivered Med-Diet-based nutritional intervention in preventing atrial tachyarrhythmia recurrence post-catheter ablation in AF patients (17). The intervention, utilizing phone contacts, web-based tools, and resource access, enhanced adherence to the Med-Diet, notably resulting in positive dietary habit changes but failed to demonstrate an effect on AF recurrence post ablation. By recognizing diet as a crucial element of lifestyle modification, tools to optimize adherence could play a pivotal role in reducing AF risk. The study underlines the benefits of the predominantly plant-based traditional Mediterranean diet, focusing on fish, olive oil, nuts, fruits, and vegetables. Intriguingly in this study, the fish component of the Med-diet, rich in n-3 fatty acids, exhibits a “U” shaped curve, highlighting the delicate balance for optimal AF protection. A secondary analysis of the PREDIMED trial reveals significant AF protection with a Med-Diet supplemented with extra virgin olive oil (EVOO), emphasizing its anti-inflammatory and antioxidant properties (18). Other studies have evaluated the Med-Diet's intersection with metabolites in the tryptophan-kynurenine pathway, which are associated with heart failure and AF risk (19) The Med-Diet, especially when supplemented with EVOO, demonstrates potential counteraction against these metabolites, emphasizing its role in regulating inflammation (18). The comprehensive PREDIMED study showed a 30% reduction in cardiovascular events, improved blood pressure, insulin resistance, and lipid profiles, emphasizing its multifaceted impact (20). Additionally, Med-Diet has a positive impact in weight loss, triglycerides, blood pressure, and diabetes mellitus, presenting a holistic approach to cardiovascular health and hence offering a dietary formula to decreasing the various risk factors involved in the development of AF. A complementary study from 2014 further supports the cardiovascular benefits of the Med-Diet, highlighting its association with reduced platelet activation and thromboxane A2 production in AF patients (21). This study, focusing on elderly AF patients at high risk of atherosclerosis and thromboembolism, establishes a link between higher Med-Diet adherence and diminished platelet activation, supported by a reduction on thromboxane B2 biosynthesis (22). Notably, wine and olive oil consumption, integral to the Med-Diet by some definitions, were independently associated with lower platelet activation levels, suggesting a beneficial effect. While the study's observational nature and limited sample size pose constraints, it introduces a novel biological explanation for the cardiovascular advantages of Med-Diet, particularly when enriched with extra virgin olive oil. Alcohol consumption, nevertheless, has been associated with higher incidence of AF as discussed later in this review and should probably be excluded for now from the Med-Diet recommendations for patients with AF. In summary, the Mediterranean Diet has been described as a nutritional powerhouse, rich in antioxidants and displaying favorable metabolic effects. Its protective role in preventing AF still needs to be further evaluated but evidence supports that the diet addresses risk factors associated with metabolic syndrome which underscores the overall value of dietary interventions in promoting cardiovascular health. The nuanced relationships between specific dietary components, remote nutritional interventions, and metabolites further emphasize the need for additional research, including larger and better controlled interventional studies, to explore the clinical impact of the Med-Diet in AF patients and its potential role in reducing cardiovascular events and disease progression. Funding and scale for such studies remains a challenge. Currently, its potential benefits and lack of evidence for harm supports a cautious recommendation in favor of the Mediterranean Diet, excluding regular alcohol intake, as a healthy alternative for patients at risk or with AF. Future research may help optimize the elements of the diet, confirm, reject or identify specific populations more likely to benefit from this recommendation.

### DASH diet

Another dietary intervention that has shown positive effect on the management of hypertension and statistical association with improved survival is the DASH diet but an effect on incidence, recurrence or progression of AF has not been demonstrated. In the Dietary Approaches to Stop Hypertension (DASH) study (23), a diet rich in fruits and vegetables combined with low-fat dairy foods in addition to reduced total and saturated fat resulted in significant blood pressure lowering effect and good adherence. Subsequent large population studies have shown an association between DASH and similar diets with improved cardiovascular outcomes and decreased mortality (24) but once corrected for other lifestyle factors have not shown a definitive effect on AF (25). Considering the effects on blood pressure, cardiovascular health, mortality and weight, it is reasonable to consider DASH as a healthy dietary alternative to recommend for patients with AF particularly those with associated hypertension and diabetes.

### Low-carbohydrate diet

Diabetes mellitus, a complex metabolic disorder, constitutes a significant contributor to the heightened risk of AF, with studies indicating a 40% increase in AF risk associated with diabetes (26). The risk escalates with higher hemoglobin A1c levels and prolonged diabetes duration, establishing a direct correlation between diabetes severity and AF susceptibility. The intricate pathophysiologic mechanisms associated to diabetes exert their effect not only directly but also indirectly, intertwining with other AF comorbidities like obesity and dietary habits. Aggressive diabetes control is recommended as part of the comprehensive lifestyle modification intervention that proved successful in the management of AF in the ARREST AF trial (27). Surprisingly and complicating our understanding, a large prospective cohort study (ARIC study) spanning over two decades unravels a novel association between dietary choices and AF incidence (28). The research identifies a higher risk of incident AF linked to a low-carbohydrate intake as a percentage of energy. This marks a departure from traditional dietary assessments primarily focused on factors like omega-3 fatty acids. The study's robust design, involving a large community-based cohort with extensive follow-up and thorough statistical adjustments, underscores the reliability of its findings. The inverse relationship between carbohydrate intake and incident AF prompts considerations about the potential mechanisms at play. The study posits that low-carbohydrate diets lead to reduced intake of anti-inflammatory foods, trigger oxidative stress, and potentially elevate the risk of other cardiovascular diseases, all of which are established risk factors for AF. However, the study also acknowledges limitations inherent in its observational nature, including potential measurement errors in dietary assessments and challenges in accurately classifying AF types. Despite these caveats, the findings highlight the cautious evaluation of low-carbohydrate diets and their implications on arrhythmia. The call for additional research, including randomized controlled trials, echoes the need to delve deeper into the intricate relationship between dietary choices, metabolic conditions like diabetes, and the multifaceted landscape of AF risk factors. Further exploration is essential to guide recommendations and interventions for primary AF prevention in the complex interplay between metabolic health and dietary patterns.

### Alcohol

Despite moderate alcohol intake being considered in the past an element of the Mediterranean Diet and a potential protective intervention against coronary artery disease, a meta-analysis conducted in 2010 consistently showed a clear association between alcohol consumption and the risk of AF onset across different settings, with varying strengths of association (29). A dose-response relationship between daily alcohol intake and AF risk was observed, suggesting a potential threshold under which the increased risk of AF may not be significant. Temporal analyses and interventions indicate the reversibility of AF following changes in alcohol consumption. The proposed mechanism is the effect of alcohol on the atrial tissue and its electrical properties, leading to abnormalities such as decreased conduction velocity and shortened refractory periods, promoting the development of atrial re-entry and the various pathophysiologic mechanism underlying AF (30). Another proposed pathophysiology mechanism involves affecting histamine levels and cytosolic sulfotransferases. Additionally, alcohol-related hypertension may contribute to atrial remodeling, further increasing the risk of AF onset (31). Both clinical and pathophysiological evidence strongly suggest that regular alcohol consumption may cause AF. A multicenter randomized controlled trial aimed to investigate the impact of reducing alcohol consumption on AF recurrence among regular drinkers with symptomatic AF found that substantial reduction in alcohol intake was associated with a decrease in AF recurrence and a reduced proportion of time spent in AF (32). Previous studies have shown a dose-related increased risk of incident AF with alcohol consumption, even with low levels of intake. However, limitations include relying on patient-reported alcohol quantities subject to recall bias, potential confounding factors, and challenges assessing secondary outcomes such as cardiovascular events. Gender variations are evident regarding the link between moderate alcohol intake and AF, with males showing a more pronounced increase in risk (33). A Danish study found that increasing alcohol intake over five years correlated with higher AF risk, but reducing intake did not significantly reduce risk (29). Overall, it has been recognized that regular alcohol consumption is a modifiable risk factor for AF and reducing alcohol intake might lead to a reduction in AF burden and recurrence. Prospective data on alcohol changes and AF risk is sparse. Limitations include self-reported alcohol intake, possible selection bias, and lack of consideration for binge drinking and sleep apnea. Observational data cannot establish causality, and AF assessment has frequently relied on diagnosis codes, possibly underestimating incidence. Based on current evidence alcohol abstinence is recommended to prevent AF and AF recurrences.

### Gluten

Celiac disease (CD) is a chronic gastrointestinal inflammatory disorder characterized by malabsorption in individuals sensitive to gluten-containing grains. While its global prevalence in the general population is around 1%, it is notably higher in patients with autoimmune disorders, reaching 8%–20% (34). CD has been linked to a significantly higher risk of major adverse cardiovascular events, including cardiac arrhythmias (35). Cardiovascular disease is the most common cause of death among these patients (36). Theoretically, inflammation and fibrosis play a significant role in developing AF (37). Studies have shown a slightly elevated risk of AF in patients with CD, both before and after the diagnosis; however, the risk was higher around the time of the diagnosis, suggesting a role of increased amount of inflammation aggravating both CD as well as AF (38). Studies have found a link to various inflammatory markers like high-sensitivity C-reactive protein, sICAM-1, and fibrinogen (38, 39). Furthermore, cases of ventricular arrhythmia in CD patients with autoimmune myocarditis improved with a gluten-free diet, indicating a potential link between CD and arrhythmias in general (40). Our group has reported a small case series of patients with gluten sensitive arrhythmias including AF and idiopathic premature ventricular contractions with near resolution of symptoms after adopting a gluten free diet (41). In summary, there is a link between autoimmune diseases and the risk of development of AF, particularly in patients with CD; however, once the confounding factors are adjusted, the risk remains only small at around 30% (38). An evaluation to rule out sub-clinical or undiagnosed CD as well as an empiric trial of gluten avoidance in patients with idiopathic or “lone” AF may be reasonable based on its simplicity and lack of harm.

### Caffeine

Contrary to common believe and the sporadic anecdotal association of caffeine intake to cardiac arrhythmias, a 2016 cohort study found that coffee ingestion and total caffeine intake were associated with a reduced risk of developing AF across various risk groups (42). While caffeine has been extensively studied, coffee contains numerous compounds besides caffeine that may contribute to health effects (43). Some studies suggest that compounds in coffee may counterbalance adverse effects of caffeine, with potential benefits for cardiovascular health. However, the specific compounds responsible for these benefits remain unidentified (43, 44). In a meta-analysis, participants consuming higher levels of coffee showed a lower risk of AF, consistent with prior findings (44). However, the study had limitations, such as potential residual confounding and the inability to distinguish between caffeinated and decaffeinated coffee intake. Despite these limitations, the study's strengths included a large sample size, detailed data collection, and almost complete adherence to follow-up. A 2018 meta-analysis concluded again that caffeine does not increase the risk of AF (45). It demonstrated an association between higher caffeine intake and a lower incidence of AF. A Potential mechanism for caffeine's protective effect against AF includes its lack of acute arrhythmogenicity in healthy individuals (44). Other studies have also found favorable outcomes associated with caffeine intake or coffee consumption, including reduced risk of death from various causes and no relationship between chronic caffeine consumption and ventricular ectopy (46). In another prospective study involving healthy middle-aged women, caffeine use was not associated with an increased risk of AF (47). Women in the highest quintile of caffeine intake had a similar AF risk to those in the lowest quintile, with minimal changes after multivariable adjustment. Interestingly, women in the third quintile of caffeine consumption were found to have lower risk of AF, suggesting the potential benefits of moderate caffeine intake. These findings, as mentioned earlier, suggest that increased caffeine consumption does not contribute to the increasing burden of AF in the general population, and moderate caffeine intake may even have a protective effect. Further studies are needed to elucidate coffee's cardioprotective effects beyond caffeine and determine potential differences in sensitivity between coffee and pure caffeine intake. The current body of evidence supports the conclusion that caffeine consumption does not increase AF incidence and could even reduce it, particularly with moderate coffee consumption. Nevertheless, most of this favorable data derive from large population studies. It remains unclear if a sub-group of individuals could be particularly sensitive to caffeine, subjects among whom caffeine intake could lead to AF or AF recurrences, acting perhaps as a trigger. In clinical practice, few patients report this clinical association and among them it remains reasonable to recommend avoidance of caffeine once identified as their reproducible trigger, regardless of the mechanism involved.

### Chocolate

Chocolate consumption in its association with AF has yielded inconsistent findings across various studies (48). A Danish population-based cohort study involving 55,502 participants over an average of 13.5 years revealed a significant association between chocolate intake and reduced AF risk (49). However, two prior studies, the Women's Health Study and a cohort study of US male physicians, found no statistically significant associations between chocolate consumption and AF risk (50, 51). The Women's Health Study, with more than 33,000 female participants followed for over 14 years, showed non-significant hazard ratios across quintiles of chocolate consumption, except for the third quintile (50). Similarly, the Physicians' Health Study, following 18,819 US male physicians for approximately nine years, revealed non-significant hazard ratios for various levels of chocolate consumption (51). Data from two cohort studies and a meta-analysis, including 180,454 participants, also found no evidence of an association between chocolate consumption and AF risk (50). In an analysis of two prospective multicenter Swiss AF cohort studies (Swiss-AF) and (BEAT-AF), chocolate consumption was found to have no association with major adverse cardiac events such as ischemic stroke, myocardial infarction or cardiovascular death in a patient population with AF (52). While some studies have shown a beneficial link between moderate chocolate consumption and other cardiovascular diseases like ischemic heart disease, heart failure, and stroke, the impact on AF appears to be neutral (53). This discrepancy could be due to the cardiovascular effects of cocoa products, such as improved endothelial function and modest reductions in blood pressure and insulin resistance, which may have less influence on AF than atherosclerosis-related cardiovascular diseases (54). Strengths of the Swedish cohort studies included their large sample sizes, adjustment for major potential confounders, and reliance on objective data from the Swedish Patient Register. However, limitations such as inevitable misclassification of chocolate consumption, lack of information on milk chocolate vs. dark chocolate consumption, and the observational design should be noted. Despite the large sample size in the meta-analysis, no association between chocolate consumption and AF risk was observed. No definitive recommendation can be made regarding chocolate consumption in relationship to AF.

### Salt

In a large-scale prospective observational study involving 473,080 adults, the relationship between estimated daily salt intake and the risk of new-onset AF was investigated (55). The study utilized urinary sodium excretion as a proxy for dietary salt intake, revealing a U-shaped association between sodium intake and AF risk among men; very low and high estimated daily sodium intakes were associated with elevated AF risk. Among women, while there was initially a tendency for a J-shaped association between sodium excretion and AF risk, this trend vanished after adjusting for established cardiovascular risk factors. A Finnish study, albeit smaller in scale, supported the association between high salt intake and increased AF risk but did not report the association between low sodium intake and AF risk among men (56). Other investigations have provided additional insights into the complex interplay between sodium intake and cardiovascular health, including its potential role on the pathophysiology of atrial tachycardia and atrial fibrillation, not solely from hypertension but also by elevating intracellular calcium levels within cardiac tissue via the sodium/calcium exchange mechanism. This elevation subsequently influences intracellular calcium release from the sarcoplasmic reticulum, consequently contributing to arrhythmias. Moreover, sodium can alter the mechano-electrical dynamics of the myocardium, potentially precipitating arrhythmias due to modifications in cell length or tension (57, 58). On the contrary, a meta-analysis of more than 1.4 million participants in 2021 indicated that salt intake does not correlate with a heightened risk of developing new-onset AF and that factors beyond salt intake itself may exert a more significant influence on the occurrence of new-onset AF (59). However, this study had some significant limitations. It combined studies with different designs, such as Mendelian randomization and cohort studies, which could lead to methodological heterogeneity. Additionally, observational studies, including Mendelian randomization studies, may suffer from selection bias as they typically recruit survivors, potentially missing individuals who have died due to cardiovascular disease related to salt intake. This could lead to a false null association between salt intake and AF risk due to competing risks rather than indicating no relationship between salt intake and AF risk. To summarize, most studies have found a strong link between salt intake and AF risk; low sodium intake is also associated with better blood pressure control and better cardiovascular outcomes which in turn may result in lower risk of AF. However, further research is warranted to elucidate causality and better understand sodium intake's role in AF development, prevention and management.

### Antioxidants and micronutrients

#### Carotenoids

Low plasma concentrations of lutein and zeaxanthin are associated with a nearly twofold increased risk of AF (60). However, carotenoids such as b-cryptoxanthin, lycopene, a-carotene, b-carotene, and total carotenoid deficiency did not significantly correlate with AF risk. Recurrent AF was used in the risk analysis, with a high incidence observed despite antiarrhythmic therapy. It emphasizes the role of inflammation and oxidative stress in AF development and suggests that carotenoids, known for their antioxidant properties, may mitigate these factors. The anti-inflammatory and antioxidant effects of carotenoids may contribute to the remodeling of atrial myocytes, reducing the risk of AF. Carotenoids, in combination with different antioxidants found in fruits and vegetables, may have synergistic effects, providing more significant health benefits than individual antioxidants. With their reported interactions in the human body by scavenging free radicals, carotenoids may independently contribute to a decreased risk of AF. In conclusion, the low plasma levels of lutein and zeaxanthin are associated with an elevated risk of AF in the elderly. It supports the idea that consuming foods rich in carotenoids like fruits and vegetables as emphasized in the Mediterranean and DASH diets may be protective against AF.

#### Flavonoids

The postulated potential benefits of flavonoids in reducing AF risk remain inconsistent. In a large Danish cohort study spanning 23 years, higher habitual intake of total flavonoids was not significantly associated with lower incident AF risk overall. However, intriguingly, flavonoid intake showed a protective effect in smokers and heavy alcohol consumers (61). Flavonoids have been implicated in mitigating cardiovascular disease markers through anti-inflammatory and anti-thrombogenic pathways, but their role in AF prevention remains uncertain (61). Notably, the observed association between flavonoid intake and reduced AF risk in specific subgroups suggests potential protective effects mediated through inflammatory and oxidative stress pathways rather than direct antiarrhythmic properties (62). While studies have provided valuable insights, limitations inherent to observational research, such as unmeasured confounders and potential changes in dietary habits over time, warrant cautious interpretation of the results. Further research is needed to authenticate these findings and explore the potential benefits of flavonoid-rich foods, particularly in high-risk populations such as smokers and heavy alcohol consumers, for AF prevention.

### Magnesium

A study involving 3,530 participants from the Framingham Offspring Study investigated the association between serum magnesium levels at baseline and the risk of atrial fibrillation in individuals free of cardiovascular disease (63). Using Cox proportional hazard regression analysis and adjusting for various factors, including conventional AF risk factors, the researchers found that lower serum magnesium levels were moderately associated with a higher risk of developing AF over a follow-up of up to 20 years. Individuals in the lower quartile of serum magnesium were approximately 50% more likely to develop AF than those in the upper quartiles. The results remained consistent even after excluding individuals on diuretics from the analysis. Given the common occurrence of hypomagnesemia in the general population, this research has potential clinical implications. Additionally, in critically ill patients, where post-operative AF is common, magnesium supplementation has shown promise in preventing AF development, likely through its anti-inflammatory and anti-arrhythmic effects (64). However, limitations such as small sample sizes, high risk of bias in some studies, and heterogeneity in outcomes should be considered when interpreting the findings. Further research is needed to validate these results, especially in critically ill populations, to improve patient outcomes and reduce healthcare utilization costs associated with AF.

### Potassium

Hypokalemia has been associated with higher risk of AF (65, 66), cardiovascular events, stroke and mortality in the general population (67, 68) and among patients with hypertension (69). It is a recognized risk factor for AF after cardiac surgery (70). Low potassium has been shown to reduce sinoatrial node activity and increase pulmonary vein firing in animal models (71). Potassium-rich foods seem to counteract the negative effect of sodium by promoting diuresis and reducing aldosterone secretion (72). Surprisingly, potassium supplementation has failed to demonstrate a reduction in the incidence of AF after cardiac surgery (73). On the other hand, the use of potassium chloride supplementation and combination of sodium and potassium chloride as dietary substitutes for sodium has been shown to have a positive effect on hypertension, cardiovascular events and death (74–77). Direct evidence on the effects of potassium supplementation in AF is still lacking. Extrapolating from its beneficial effects on hypertension and cardiovascular outcomes and evidence in favor of the Mediterranean and DASH diet, it is reasonable to consider the recommendation of potassium-based salt substitutes for patients with or at risk of AF. Caution and close monitoring must be considered amongst patients with renal function impairment and at risk for hyperkalemia.

### Miscellaneous micronutrients

Deficiencies and levels of certain other micronutrients, such as selenium (Se) and iron (Fe), have been linked to new-onset atrial fibrillation in a large community-based cohort. After adjusting for potential confounders, Se deficiency, similar to Mg deficiency, was associated with an increased risk of new-onset AF, particularly in non-smoking participants (78). Se deficiency also showed a significant association with non-smoking participants experiencing over a 65% increased risk. Mechanistically, it may contribute to mitochondrial dysfunction and oxidative stress, two factors involved in the pathogenesis of AF and heart failure. However, Fe deficiency did not show a significant association with new-onset AF, although it was observed that older men had a slightly higher risk (78). Histamine, derived from histidine, plays a role in immune responses and cardiovascular regulation. Elevated levels are associated with arrhythmogenic potential, stimulating H2 receptors to accelerate heart rate and trigger diastolic depolarization, potentially leading to atrial tachycardia (79). Histamine-induced depolarization of Purkinje fibers may promote ventricular tachycardia (79). Rare cases link hyper-histaminemia to cardiac arrests and AF (80). Due to its wide range of pro-arrhythmic properties, a pilot study suggested elevated levels in 21.2% of AF patients, possibly due to histamine-containing foods, allergies, infections, or immune disorders (81). However, prospective data on histamine's role in AF are limited. These findings suggest that nutritional imbalances may represent modifiable risk factors for AF, independent of heart failure development, highlighting potential avenues and research for AF prevention.

### Obesity and atrial fibrillation

Obesity is prevalent in the US, where both obesity and AF burdens are high. The Long-Term Effect of Goal-Directed Weight Management in an Atrial Fibrillation Cohort: A Long-Term Follow-Up Study (LEGACY) study, focusing on 355 overweight AF patients, demonstrated the remarkable impact of modest weight loss (82). During the waiting period for their AF ablation procedure, 38% successfully lost around 36 pounds, resulting in significant AF reduction. Nearly half of the patients achieved complete remission solely through weight loss, eliminating the need for antiarrhythmics or ablation. Moreover, improvements were observed in systolic blood pressure, C-reactive protein, diabetes remission, LDL levels, triglycerides, echocardiographic abnormalities, and overall sense of well-being. Obesity's intricate relationship with AF involves complex mechanisms, including inflammation and oxidative stress, with weight loss showing promise in reducing AF development and recurrence (83). Similar reductions in AF have been reported among patients undergoing bariatric surgery (84). Challenges remain in understanding variations in AF risk associated with different fat types and the impact of obesity on permanent AF. Despite the mainstream belief in weight loss benefits, a post hoc analysis of the Atrial Fibrillation Follow-up Investigation of Rhythm Management (AFFIRM) study demonstrated an “obesity paradox” with better clinical outcomes amongst obese patients with AF (85). On the other hand, early-life obesity has been recognized as a predictor of AF risk. Epicardial fat, a key player in left atrial remodeling, poses imaging challenges when quantifying it to establish its relationship with AF severity. Based on the overall benefits of weight reduction in cardiovascular health, blood pressure and the results of the LEGACY (82) and similar clinical studies, weight reduction is recommended for reduction in the burden of AF and as co-adjuvant to antiarrhythmic drug therapy and catheter-based ablation.

### Bacteriome, diet and atrial fibrillation

Multiple studies have demonstrated a strong association between alterations in the gut microbiota and the risk of cardiovascular disease and AF. It is described that patients with AF develop dysbiotic gut microbiota with higher microbial diversity and specific composition patterns. It is recognized that diet and drugs could be important determinants in the composition of the gut bacteriome (22). Bacteria metabolites like gut-derived lipopolysaccharide, Trimethylamine N-Oxide, secondary bile acids (86–89) are found elevated in patients with AF and linked to atrial inflammation and adverse electrical remodeling. Meanwhile the gut microbiota changes accompanying AF are also associated with disrupted production of the rather protective short chain fatty acids (90). Fecal transplantion studies in animals has demonstrated that transplanting dysbiotic microbiota or microbiota from aged animals to healthy subjects results in higher susceptibility to AF, increased levels of circulating lipopolysaccharide and evidence of inflammation and fibrosis in the atrium (91, 92). Despite the current body of evidence supporting an association between AF, the gut microbiota and its derived metabolites, it is not clear if they have causal or modulation effect. Even less understood is the potential opportunity to intervene via diet, pharmacotherapy or fecal transplantation in the prevention or treatment of AF (93).

## Proposed mechanisms

AF is a complex arrhythmia with multiple underlying mechanisms. Several dietary factors and micronutrients studied for their potential role in the pathophysiology of AF affect various of these mechanisms, as described in this article. There is an overlap between the different mechanisms outlined here, but it is important to recognize the contrast between these separate processes and their respective significance in the complex pathophysiology of this arrythmia. Following is a brief overview of the various proposed mechanisms of action mediating the interactions between diet and the pathophysiology, clinical manifestation and progression of AF.

### Inflammation and oxidative stress

Oxidative stress, caused by an imbalance between reactive oxygen species (ROS) production and the body's antioxidant defenses, is significantly implicated in AF development and remodeling (94). Studies have demonstrated that increased oxidative stress in atrial tissue leads to protein modifications and calcium accumulation, contributing to AF by reducing antioxidant levels like vitamin C and glutathione (95). Key sources of ROS in AF include NADPH oxidase, xanthine oxidase, nitric oxide synthase uncoupling, myeloperoxidase, and monoamine oxidase. These enzymes, particularly NADPH oxidase, are activated by conditions such as hypertension and hyperglycemia, leading to fibrosis and atrial remodeling. Experimental evidence also points to mitochondrial dysfunction and ROS production causing calcium leaks that trigger AF. Myeloperoxidase and monoamine oxidase further contribute to atrial fibrosis and oxidative damage, exacerbating AF risk. Ultimately, excessive ROS alter ionic currents and cellular signaling, prolonging action potentials, reducing cardiac conduction, and promoting re-entry and focal activity, which are central to AF pathogenesis. Diets rich in antioxidants, like fruits, vegetables, and whole grains, may help mitigate inflammation and oxidative stress. Micronutrients like vitamins C and E and polyphenols found in foods like berries and green tea have antioxidant properties and may reduce AF risk by combating oxidative damage (62). As previously mentioned in this review, the Mediterranean diet and a diet rich in carotenoids may mitigate the development and progression of AF through their antioxidant properties. Coffee has also been linked to decrease inflammation as it contains high levels of antioxidants such as cafestol, polyphenol, trigonelline, chlorogenic acid, and quinine. Moderate coffee consumption, through the aforementioned mechanism, has been linked to decrease incidence and risk of developing atrial fibrillation (44).

### Electrical and structural remodeling

AF is associated with electrical as well as structural remodeling of the atria, characterized by alteration in ion channel function and atrial conduction. Omega-3 fatty acids found in fatty fish, like salmon and mackerel, have been shown to modulate ion channel function and stabilize atrial electrical activity, potentially reducing susceptibility to AF (96). This mechanism may be shared with other nutrients that prevent atrial inflammation, mechanical stress and cellular uncoupling. Structural changes in the atria, such as fibrosis and hypertrophy, contribute to AF substrate formation. Diets low in sodium help mitigate atrial fibrosis by reducing inflammation, hypertension and oxidative stress (97). Hyperglycemia and foods rich in sodium have been linked to atrial fibrosis, left atrial enlargement, and cause electrical and autonomic remodeling which can lead to inter and intra-atrial conduction delays. Potassium-rich foods like bananas, potatoes, and avocados may counteract the pro-fibrotic effects of sodium by promoting diuresis and reducing aldosterone secretion (72).

### Autonomic nervous system dysfunction

Imbalances in the autonomic nervous system, both sympathetic and parasympathetic overactivity, can promote AF initiation and maintenance (98). In patients with established AF or structural heart abnormalities, sympathetic stimulation may be the driving factor in AF initiation (99). Vagal stimuli such as eating, sleeping, relaxation, and alcohol consumption have been identified as triggers for AF, particularly in younger individuals or those with a family history of AF (100, 101). Alcohol has been linked to shortened effective refractory period and low alcohol consumption may help reduce sympathetic tone and lower AF risk (33). Also, alcohol consumption has been linked to increased vagal stimulation and increasing the incidence of AF (101). This was more commonly reported in younger individuals and with beer and red wine consumption. Additionally, magnesium, present in foods like nuts, seeds, and leafy greens, plays a role in regulating autonomic function and may have antiarrhythmic effects with low magnesium levels been linked to increased automaticity (63). There have been multiple reports of vagal AF induced by ingestion of cold beverages as well (102). Vagal triggers for AF, such as eating or occurrences solely at night without adrenergic triggers, have been reported by a significant proportion of patients with paroxysmal AF (103).

### Cell membrane stability

Deficiencies in certain micronutrients, such as magnesium and potassium, have been implicated in AF pathogenesis. By its effect on the slow-activating delayed rectifier K channel (IKs) and calcium channels (L-type) in the atria, Magnesium can stabilize the cardiac cell membrane and play a protective role against AF (104). PUFAs stabilize cardiomyocyte membranes and their integration into the cell membrane's phospholipids alters ionic currents, such as the sodium and calcium channels, leading to antiarrhythmic effects (105). Chronic administration of PUFAs results in membrane incorporation that modifies ion channel behaviors and reduces arrhythmogenic activity. By inhibiting atrial-specific potassium currents and decreasing Na+ -Ca2+ exchange current, it reduces delayed after depolarizations and arrhythmia risk. Balanced diets containing adequate amounts of these nutrients, along with supplementation, when necessary, may help maintain cardiac electrical stability and reduce AF susceptibility (64).

**Figure 1:**
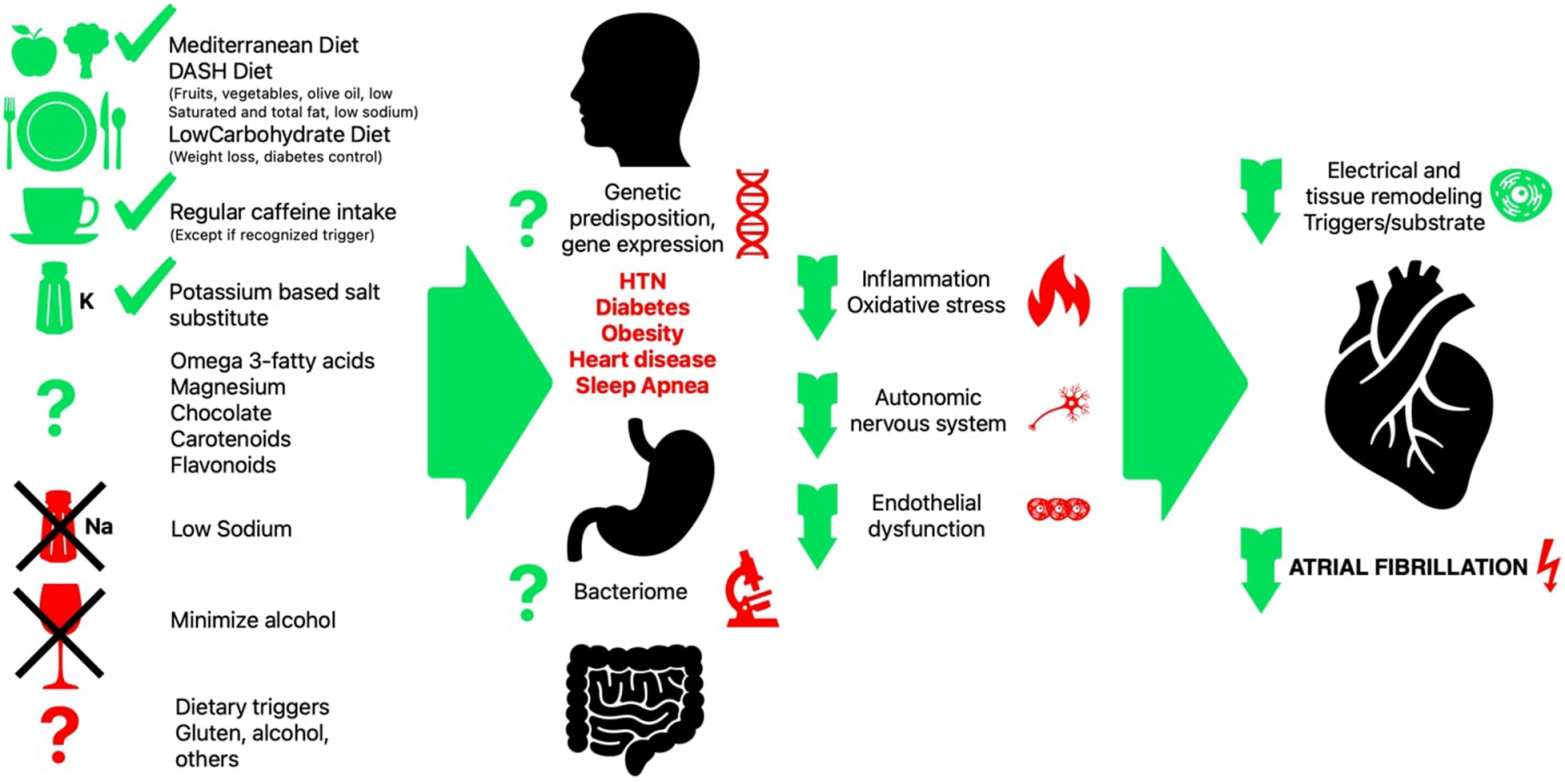
Graphic representation of recognized positive and negative dietary risk factors and their interaction with individual non dietary risk factors. The poorly understood contribution of some dietary components, triggers, genetic predisposition and gut bacteriome is represented with question marks.

In summary, various dietary factors and micronutrients can influence multiple mechanisms involved in AF pathogenesis, including inflammation, oxidative stress, electrical and structural remodeling, autonomic dysfunction, and metabolic abnormalities (Figure 1). Adopting a balanced and nutrient-rich diet may reduce AF risk and improve outcomes. However, individual dietary strategies should be tailored based on specific patient characteristics and influenced by potentially identifiable individual underlying mechanisms contributing to AF.

## Highlighted studies

Table 1.

**Table 1:** Overview of some of the important and highlighted articles.

Title	No. of patients	Result	Author name, publication year	PMID/reference no.
Fish intake and risk of incident atrial fibrillation	4,815	Consuming tuna or other broiled or baked fish was linked to a lower risk of AF, likely due to long-chain n-3 fatty acids’ beneficial effects on cardiovascular health. However, no similar benefit was observed with fried fish or fish sandwiches, possibly due to differences in nutrient composition.	Mozaffarian, 2004	15262826
Effect of Marine Omega-3 Fatty Acid and Vitamin D Supplementation on Incident Atrial Fibrillation: A Randomized Clinical Trial	24,127	Supplementation with marine omega-3 fatty acids and/or vitamin D3 did not significantly affect the incidence of AF over a median of 5.3 years. The results indicate no benefit or major risk associated with these supplements for AF prevention, suggesting that neither omega-3 fatty acids nor vitamin D3 should be used for the primary prevention of AF.	Albert, 2021	33724323
Omega-3 Fatty Acid Biomarkers and Incident Atrial Fibrillation	7,720	Higher levels of circulating and tissue omega-3 fatty acid biomarkers were not linked to an increased incidence of AF, suggesting that findings from high-dose omega-3 supplementation trials in cardiovascular disease populations may not apply to lower habitual dietary intakes.	Qian, 2023	37468189
Dietary Fish and Long-Chain n-3 Polyunsaturated Fatty Acids Intake and Risk of Atrial Fibrillation: A Meta-Analysis	12,913	This meta-analysis found no overall association between higher fish consumption or intake of n-PUFAs and AF development. However, a cautious interpretation is advised regarding very high PUFA intake showing a potential increased risk of AF, which was sensitive to individual studies. Despite the known cardiovascular benefits of n-3 PUFAs, this meta-analysis suggests that dietary guidelines recommending fish consumption for general cardiovascular health should continue, though their impact on AF prevention remains uncertain.	Li, 2017	28850090
Prevention of recurrent arrhythmias with Mediterranean diet (PREDIMAR) study in patients with atrial fibrillation: Rationale, design and methods	720	The PREDIMED trial provides strong evidence for the Mediterranean diet enriched with extra EVOO as an effective strategy for primary AF prevention, likely due to its anti-inflammatory and antioxidant properties. Managing cardiovascular risk factors through diet and lifestyle modifications can reduce the burden and severity of AF, lowering the incidence and recurrence post-ablation.	Barrio-Lopez, 2020	31809992
Extravirgin olive oil consumption reduces risk of atrial fibrillation: the PREDIMED trial	6,705	In this secondary analysis of the PREDIMED trial, a Mediterranean diet enriched with EVOO was found to significantly reduce the relative risk of AF by 38%. The strong anti-inflammatory and antioxidant properties of EVOO, attributed to its phenolic compounds, likely explain its protective effect against AF, highlighting the potential of the MeDiet with EVOO for primary AF prevention.	Barrio-Lopez, 2020	24787471
A Remote Nutritional Intervention to Change the Dietary Habits of Patients Undergoing Ablation of Atrial Fibrillation: Randomized Controlled Trial	720	A remote nutritional intervention inspired by the PREDIMAR trial effectively enhanced participants’ knowledge, skills, and adherence to a Mediterranean diet. Additionally, the study suggests that such remote health promotion interventions could be a cost-effective strategy for mitigating the growing health burden.	Goni, 2020	33284131
A clinical trial of the effects of dietary patterns on blood pressure. DASH Collaborative Research Group	459	This trial showed that a diet rich in fruits, vegetables, and low-fat dairy products, with reduced saturated and total fat, significantly lowered blood pressure in adults, demonstrating an effective non-pharmacological approach for preventing and treating hypertension, with implications for reducing cardiovascular disease risk across the population.	Appel, 1997	9099655
Mediterranean, DASH, and Alternate Healthy Eating Index Dietary Patterns and Risk of Death in the Physicians’ Health Study	15,768	In this study of US male physicians, higher MED, DASH, and AHEI diet scores were inversely associated with total mortality. The benefits of these dietary patterns on mortality may be due to the cohort's high education level and adherence to optimal medical treatments and dietary recommendations, as well as potential biological mechanisms such as improved inflammation, vascular function, and glucose-insulin homeostasis.	Patel, 2021	34072912
Associations of dietary patterns, ultra-processed food and nutrient intake with incident atrial fibrillation	121,300	This observational study found that higher consumption of ultra-processed foods increased the risk of incident AF. While adherence to Mediterranean-style and DASH diets initially appeared to reduce AF risk, these associations lost significance after adjusting for BMI and lifestyle factors, suggesting that focusing on modifiable risk factors like weight may be more crucial than specific dietary patterns for preventing AF.	Tu, 2023	37460193
Low-Carbohydrate Diets and Risk of Incident Atrial Fibrillation: A Prospective Cohort Study	13,385	In this study, low-carbohydrate intake was associated with a higher risk of incident AF, independent of other risk factors. This adverse association was consistently observed in multiple sensitivity analyses, suggesting that the increased risk may be due to reduced intake of anti-inflammatory foods and increased oxidative stress. This study is the first to explore the relationship between carbohydrate intake and AF risk, highlighting the potential long-term cardiovascular risks of low-carbohydrate diets.	Zhang, 2019	31020911
Five-year changes in alcohol intake and risk of atrial fibrillation: a Danish cohort study	43,758	This study found that increasing high alcohol intake over five years was linked to a higher risk of AF compared to maintaining a low or moderate alcohol intake. On the other hand, reducing alcohol intake did not significantly alter the risk of AF compared to stable consumption levels. These findings indicate that to prevent AF, it is advisable to avoid increasing alcohol consumption.	Frederiksen, 2023	36508613
Alcohol Abstinence in Drinkers with Atrial Fibrillation	140	AF is common, and alcohol consumption can influence its occurrence. This study showed that significantly reducing alcohol intake in regular drinkers with symptomatic AF led to fewer AF recurrences and less time spent in AF. Regular alcohol consumption is a modifiable risk factor for AF and reducing intake from an average of 17 drinks per week to 2 drinks per week can decrease AF burden and recurrence risk.	Voskoboinik, 2020	31893513
Alcohol and incident atrial fibrillation - A systematic review and meta-analysis	n/a	Low alcohol intake does not contribute to the development of AF. However, gender differences are observed with moderate alcohol intake: men show a higher increase in AF risk than women. High alcohol intake significantly increases the risk of AF in both genders.	Gallagher, 2017	28867013
Risk of idiopathic dilated cardiomyopathy in 29 000 patients with celiac disease	144,429	This study is the first to confirm dilated cardiomyopathy (DCM) diagnosis with patient charts and echocardiography in celiac disease (CD) patients, finding a 73% increased DCM risk, especially in the first year after CD diagnosis. The study identified 17 patients with both CD and idiopathic DCM, supporting previous reports of increased DCM prevalence in CD patients. Despite some limitations, the findings suggest a possible link between CD and DCM through shared inflammation and autoimmune mechanisms.	Emilsson, 2012	23130142
Small-Intestinal Histopathology and Mortality Risk in Celiac Disease	46,121	This study examined mortality risk in CD relative to small-intestinal histopathology, finding a small but significant excess mortality risk, particularly in the first year post-diagnosis. Over 3,000 deaths were recorded among 29,000 CD patients, with an overall mortality hazard ratio (HR) of 1.39, lower than many previous studies. The study uniquely explored mortality in patients with inflammation without villous atrophy and latent CD, indicating the highest mortality risk in those with inflammation.	Ludvigsson, 2009	19755695
Risk of atrial fibrillation associated with coffee intake: Findings from the Danish Diet, Cancer, and Health study	57,053	This study found that both coffee consumption and total caffeine intake are associated with a reduced rate of incident AF, consistent across various subgroups. Coffee's health impacts may stem from its complex mixture of bioactive compounds, beyond just caffeine. Despite some limitations, the study's strengths include a large sample size and comprehensive data, supporting the potential cardiovascular benefits of coffee.	Mostofsky, 2016	26701875
Association of Coffee Consumption with Atrial Fibrillation Risk: An Updated Dose-Response Meta-Analysis of Prospective Studies	723,825	This meta-analysis found no increased risk of AF with high or moderate coffee consumption, and suggested a potential decrease in AF risk with higher coffee intake. Unlike previous analyses mixing caffeine and coffee, this study focused on pure coffee and found a possible protective effect. Despite limitations, the study's large sample size and high-quality prospective design provide robust evidence that moderate to high coffee consumption does not increase AF incidence and may even have protective benefits.	Cao, 2022	35872898
Does Caffeine Consumption Increase the Risk of New-Onset Atrial Fibrillation	176,675	Coffee consumption does not increase the incidence of AF. In fact, our findings indicate a lower incidence of AF when caffeine consumption exceeds 436 mg/day. Consequently, based on the available evidence, there is no link between caffeine intake and an increased risk of AF.	Abdelfattah, 2018	29966128
Chocolate intake and risk of clinically apparent atrial fibrillation: the Danish Diet, Cancer, and Health Study	55,502	Study found that higher chocolate intake was associated with a lower rate of clinically apparent AF among both men and women, even after adjusting for total caloric intake. This supports results from previous studies, though findings varied based on sex and methodology. The study suggests that chocolate's antioxidant, anti-inflammatory, and magnesium content may contribute to cardiovascular benefits, despite potential limitations such as unmeasured confounding factors and reliance on self-reported data.	Mostofsky, 2017	28536115
Chocolate consumption and risk of atrial fibrillation: Two cohort studies and a meta-analysis	40,009	In this study, chocolate consumption showed no association with the risk of AF after adjusting for other risk factors, a finding confirmed by a complementary meta-analysis. These results contrast with earlier observations of inverse associations between moderate chocolate consumption and the risk of ischemic heart disease, heart failure, and stroke, both in the Swedish cohorts and in meta-analyses of all available cohort data.	Larsson, 2018	29224650
Estimated salt intake and risk of atrial fibrillation in a prospective community-based cohort	473,080	This study revealed a U-shaped relationship between estimated daily salt intake and the risk of AF among men. While a potential J-shaped association in women was not statistically significant, the analyses may have lacked sufficient statistical power. These findings indicate that beyond a certain physiological minimum level, higher salt intake correlates with an increased risk of AF.	Wuopio, 2021	33210391
Salt as a Trigger for Atrial Tachycardia/Fibrillation	473,080	Excessive sodium intake, contributing to fluid retention and hypertension, is a recognized risk factor for AF. Beyond hypertension, sodium may trigger arrhythmias like AF by influencing intracellular calcium levels and altering the mechano-electrical environment of the heart through stretch-induced mechanisms. Controlled studies involving larger patient populations are crucial to fully understand salt's role as a trigger for paroxysmal atrial fibrillation.	Goddard, 2022	35891840
Urinary Sodium Excretion, Blood Pressure, and Risk of Future Cardiovascular Disease and Mortality in Subjects Without Prior Cardiovascular Disease	457,484	This study demonstrated a consistent relationship between estimated urinary sodium excretion (a marker of sodium intake) and elevated blood pressure, even when restricted to subjects without baseline comorbidities. Despite clear evidence linking sodium intake to increased blood pressure, no straightforward linear relationship between high sodium intake and increased risk of mortality or cardiovascular disease was found. These findings support public health policies advocating sodium reduction to lower blood pressure, though the long-term benefits on cardiovascular events require further investigation.	Welsh, 2019	31067194
Salt intake and new-onset of atrial fibrillation: A meta-analysis of over 1.4 million participants	1,421,826	This study demonstrated a significant correlation between genetically determined high dietary salt intake and an increased risk of AF. Future research is needed to further elucidate this relationship and confirm the generalizability of our findings across more socioeconomically and ethnically diverse populations.	Bhagavathula, 2021	33933725
Low levels of plasma carotenoids are associated with an increased risk of atrial fibrillation	1,847	This prospective cohort study found that low plasma concentrations of lutein and zeaxanthin are associated with nearly a twofold increased risk of AF, while other carotenoids were not linked to AF risk. Hypertension and oxidative stress are key factors in AF development, with carotenoids potentially reducing AF risk through their antioxidant and anti-inflammatory properties. The study suggests that a diet rich in carotenoids from fruits and vegetables may help protect against AF, especially in the elderly.	Karppi, 2013	23238698
Intake of dietary flavonoids and incidence of ischemic heart disease	54,496	In this study, no clear associations were found between total flavonoid intake and ischemic heart disease (IHD) risk. However, higher intakes of flavonols and flavanol oligo + polymers were linked to lower IHD risk among ever-smokers, but not never-smokers. These findings suggest flavonoids may offer modest protection against IHD, particularly for smokers, though further research is needed.	Parmenter, 2023	36284213
Low serum magnesium and the development of atrial fibrillation in the community: the Framingham Heart Study	3,530	In this longitudinal, community-based cohort, low serum magnesium was linked to the development of AF. This study extends the known association between low magnesium and AF risk beyond the context of cardiac surgery. The findings suggest potential public health implications, warranting further research to confirm if magnesium supplementation could reduce AF risk in other populations.	Khan, 2013	23172839
Effects of magnesium on atrial fibrillation after cardiac surgery: a meta-analysis	2,490	Magnesium administration effectively reduces postoperative AF, with an impact comparable to proposed antiarrhythmic drugs. However, it did not significantly decrease hospital length of stay or mortality. Further research is needed to determine the optimal administration regimen and its efficacy when combined with other medications.	Miller, 2005	15831645
Serum potassium levels and the risk of atrial fibrillation: the Rotterdam Study	4,059	This study demonstrated that low serum potassium levels are linked to an increased risk of AF in the general population, independent of various potential confounders. Although the proportion of cases attributable to low serum potassium may be small, this finding is significant at a population level due to the routine measurement of serum potassium and the common and serious consequences of atrial fibrillation, such as stroke. Further research with repeated measurements of serum potassium and its association with atrial fibrillation risk would be valuable.	Krijthe, 2013	24012173
Serum electrolyte concentrations and risk of atrial fibrillation: an observational and mendelian randomization study	15,792	An observational study indicated that hypokalemia, hypomagnesemia, and hyperphosphatemia are associated with the onset of AF. However, MR analysis did not confirm a causal role for serum electrolytes in the development of AF. Consequently, therapies targeting electrolyte disorders like hypokalemia, hypomagnesemia, and hyperphosphatemia to prevent AF may offer limited clinical benefit.	Wu, 2024	38493091

## Future directions and clinical implications

Future directions and clinical implications of exploring diets and micronutrients in their relationship with AF encompass several key areas, focusing on both preventive strategies and therapeutic interventions. Here are some potential future directions and their clinical implications:

### Precision nutrition approaches

Future research may delve into personalized nutrition strategies tailored to individuals based on their genetic predispositions, comorbidities, and lifestyle factors. Precision nutrition aims to optimize dietary interventions for AF prevention and management by considering individual variations in metabolism, gut microbiota composition, and dietary preferences. Clinical implementation of precision nutrition in AF management could involve comprehensive dietary assessments, genetic testing, and targeted nutritional interventions tailored to each patient's specific needs.

### Clinical trials of dietary interventions

Large-scale randomized controlled trials investigating the efficacy of specific dietary interventions in preventing or managing AF are needed. These trials should evaluate various dietary patterns (e.g., Mediterranean diet, DASH diet), individual nutrients (e.g., omega-3 fatty acids, magnesium), and dietary supplements (e.g., antioxidants, vitamin D) in diverse patient populations with AF. Clinical trials should assess the effects of dietary interventions on AF recurrence and their impact on clinical outcomes, such as AF burden, progression, stroke risk, hospitalizations, heart failure and mortality.

### Longitudinal cohort studies

Prospective longitudinal cohort studies with extended follow-up duration are essential for elucidating the long-term effects of dietary habits and nutrient intake on AF incidence and progression. To accurately capture dietary exposures, these studies should incorporate comprehensive dietary assessments, including food frequency questionnaires, 24-h dietary recalls, and biomarker measurements. Longitudinal cohorts can provide valuable insights into the temporal relationship between dietary factors and AF risk, identify potential dietary biomarkers of AF susceptibility, and elucidate the mechanisms underlying diet-AF associations. Modern tools like artificial intelligence and personal digital devices will facilitate collection, management and analysis of more detailed and accurate data points amongst larger populations.

### Mechanistic studies

Further mechanistic studies are warranted to better understand the precise pathways through which specific diets and micronutrients influence AF pathogenesis. Mechanistic investigations should explore the effects of dietary components on inflammation, oxidative stress, ion channel function, autonomic tone, cardiac remodeling and other vital mechanisms underlying AF initiation and maintenance. Advanced experimental techniques, such as cellular electrophysiology, tissue engineering, and omics approaches, can help unravel the molecular mechanisms mediating the effects of diet on cardiac electrophysiology and structure.

### Integration of nutritional counseling into AF management

Incorporating nutritional counseling and lifestyle modifications into routine AF management can optimize patient care and improve clinical outcomes. Healthcare providers should assess patients’ dietary habits, provide personalized recommendations based on evidence-based guidelines, and support behavior change. Multidisciplinary care teams, including dietitians, nurses, and pharmacists, can collaborate to deliver comprehensive nutritional interventions tailored to each patient's unique needs and preferences. Adequate monitoring and ongoing reinforcement are also recommended.

### Telehealth and digital health solutions

Leveraging telehealth and digital health platforms can facilitate not only research but the delivery of dietary counseling and monitoring for patients with AF, especially in remote or underserved areas. Telehealth platforms can enable real-time dietary tracking, remote consultations with healthcare providers, virtual support groups, and nutrition and AF management educational resources. Integrating digital health solutions into routine clinical practice can enhance patient engagement, adherence to dietary recommendations, and self-management of AF.

Future directions in exploring dietary interventions and micronutrients in AF development encompass a multifaceted approach involving precision nutrition, clinical trials, longitudinal cohort studies, mechanistic research, nutritional counseling, and digital health solutions. By advancing our understanding of the role of diet in AF pathophysiology and implementing evidence-based nutritional interventions, healthcare providers can empower patients to adopt healthy dietary habits and reduce their risk of AF-related complications.

### The HEAD-2-TOES scheme

The HEAD-2-TOES scheme was recently proposed as a comprehensive method to assist clinicians in managing and controlling specific risk factors associated with the development of atrial fibrillation (106). Controlling these modifiable risk factors is particularly crucial in the context of primary prevention. This scheme identifies and addresses various determinants, detailed in the table below, and provides an overview of both primary and secondary prevention targets. Additionally, it discusses the impact of various diets on these risk factors, highlighting how dietary choices can influence the effectiveness of prevention strategies (Table 2).

**Table 2:** HEAD-2-TOES table of various diets and their effect on atrial fibrillation.

Acronym	Risk factor	Primary prevention targets	Secondary prevention targets	Effect of various diets
H	Heart failure with reduced ejection fraction	ACE inhibitor or ARB, β-blocker, MRA, SGLT2 inhibitor	ACE inhibitor or ARB, MRA	Mediterranean diet has been shown to reduce inflammation and lower the risk of hospitalization and mortality in HFrEF patients. DASH diet can help lower blood pressure and improve heart health in HFrEF patients.
E	Exercise (physical inactivity)	≥150 min per week MVPA	≥200 min/per week MVPA	Mediterranean, DASH and high-fiber diets can reduce inflammation, lower blood pressure and decrease fluid retention, lessening the workload on the heart and help increase exercise tolerance and physical capacity of these patients.
A	Arterial hypertension	BP <130/80 mmHg	BP <130/80 mmHg (rest) and <200/100 mmHg (exercise)	Mediterranean, DASH and diets rich in Omega-3 fatty acids can reduce inflammation, decrease salt retention and decrease the strain on the heart, hence, help treat and reduce the incidence of essential hypertension and mitigates risk of AF.
D2	Diabetes mellitus type 2	HbA1c <6.5%	Dietary changes and HbA1c <6.5%	Low carbohydrate diet as well as Mediterranean, DASH and high-fiber diets can improve insulin sensitivity and reduce blood sugar levels which reduces the risk of AFib complications in people with diabetes.
T	Tobacco smoking	Complete cessation	Complete cessation	Various diets, including diets rich in antioxidants can help protect against oxidative stress caused by smoking, reducing damage to the heart and blood vessels, which can benefit those with AFib. This includes Mediterranean, DASH, Omega-3 fatty acids and diets rich in fruits and vegetables.
O	Obesity	BMI ≤25 kg/m^2^	10% weight reduction; BMI ≤27 kg/m^2^	Mediterranean, low-carbohydrate, DASH, Omega-3 fatty acids diets stabilize blood sugar levels, promote weight loss, lower blood pressure, and enhances heart health, making it beneficial for obese patients with AFib.
E	Ethanol consumption	≤1 standard drink per day	≤3 standard drinks per week	Some of the same diets as above can help provide antioxidants that help mitigate some of the negative effects of alcohol on the heart, benefiting AFib patients.
S	Sleep apnea	AHI <15	AHI <15 without CPAP; CPAP for AHI ≥30 or AHI ≥20 with hypertension	Mediterranean diet can improve cardiovascular health, reduce inflammation, and aid in weight management, which can alleviate symptoms of OSA and benefit AFib patients. SImilarly, DASH and low-carbohydrate diets can lower blood pressure, improve heart health, and promote weight loss, which can help manage both OSA and AFib.

## Conclusions

In conclusion, dietary interventions represent a promising avenue in managing AF. This review highlights the growing body of evidence supporting the role of diet in modulating AF risk and progression. A comprehensive analysis of existing literature has elucidated the potential mechanisms by which various dietary patterns, nutrients, and supplements may influence AF pathophysiology. From adopting heart-healthy dietary patterns such as the Mediterranean diet and DASH diet to incorporating specific nutrients like omega-3 fatty acids, magnesium, and antioxidants, dietary interventions offer a holistic approach to AF management. Moreover, emerging research underscores the importance of personalized nutrition strategies tailored to individual patient profiles, genetic predispositions, and lifestyle factors. Large clinical trials, longitudinal cohort studies, and mechanistic research are needed to elucidate further dietary interventions' efficacy, safety, and mechanisms in AF prevention and treatment. By integrating nutritional counseling, lifestyle modifications, and digital health solutions into routine clinical practice, healthcare providers could empower patients to optimize their dietary habits and improve their AF outcomes. Overall, dietary interventions hold promise as adjunctive therapies in the comprehensive management of AF and warrant further exploration in future research endeavors

## Important take-home points

• Dietary interventions offer a promising approach for managing atrial fibrillation, with potential in modulating AF risk and progression.
• Adopting heart-healthy dietary patterns such as the Mediterranean diet and DASH diet is particularly beneficial for AF management.
• Incorporating specific nutrients like omega-3 fatty acids, magnesium, and antioxidants can positively impact AF management.
• Emerging research underscores the importance of personalized nutrition strategies tailored to individual patient profiles, genetic predispositions, and lifestyle factors.
• Large clinical trials, longitudinal cohort studies, and mechanistic research are needed to further elucidate the efficacy, safety, and mechanisms of dietary interventions in AF prevention and treatment.
• Integrating nutritional counseling, lifestyle modifications, and digital health solutions into routine clinical practice can empower patients to optimize their dietary habits and improve their AF outcomes.

## Data Availability

Requests to access the datasets should be directed to muhammad.nabil@bswhealth.org.
